# Expression of a fungal manganese peroxidase in *Escherichia coli*: a comparison between the soluble and refolded enzymes

**DOI:** 10.1186/s12896-016-0317-2

**Published:** 2016-12-01

**Authors:** Nan Wang, Kai Ren, Rong Jia, Wenting Chen, Ruirui Sun

**Affiliations:** School of Life Science, Anhui University, 111 Jiulong Road, Economic and Technology Development Zone, Hefei, Anhui 230601 People’s Republic of China

**Keywords:** *Escherichia coli*, Inclusion bodies, Kinetic properties, Manganese peroxidase, Protein expression, Protein refolding, Spectral characteristics

## Abstract

**Background:**

Manganese peroxidase (MnP) from *Irpex lacteus* F17 has been shown to have a strong ability to degrade recalcitrant aromatic pollutants. In this study, a recombinant MnP from *I. lacteus* F17 was expressed in *Escherichia coli* Rosetta (DE3) in the form of inclusion bodies, which were refolded to achieve an active enzyme. Further, we optimized the in vitro refolding conditions to increase the recovery yield of the recombinant protein production. Additionally, we attempted to express recombinant MnP in soluble form in *E. coli*, and compared its activity with that of refolded MnP.

**Results:**

Refolded MnP was obtained by optimizing the in vitro refolding conditions, and soluble MnP was produced in the presence of four additives, TritonX-100, Tween-80, ethanol, and glycerol, through incubation at 16 °C. Hemin and Ca^2+^ supplementation was crucial for the activity of the recombinant protein. Compared with refolded MnP, soluble MnP showed low catalytic efficiencies for Mn^2+^ and H_2_O_2_ substrates, but the two enzymes had an identical, broad range substrate specificity, and the ability to decolorize azo dyes. Furthermore, their enzymatic spectral characteristics were analysed by circular dichroism (CD), electronic absorption spectrum (UV-VIS), fluorescence and Raman spectra, indicating the differences in protein conformation between soluble and refolded MnP. Subsequently, size exclusion chromatography (SEC) and dynamic light scattering (DLS) analyses demonstrated that refolded MnP was a good monomer in solution, while soluble MnP predominantly existed in the oligomeric status.

**Conclusions:**

Our results showed that two forms of recombinant MnP could be expressed in *E. coli* by varying the culture conditions during protein expression.

**Electronic supplementary material:**

The online version of this article (doi:10.1186/s12896-016-0317-2) contains supplementary material, which is available to authorized users.

## Background

Manganese peroxidase (MnP, E.C. 1.11.1.13) can be isolated from many species of white-rot fungi, and it catalyzes the oxidation of Mn^2+^ to Mn^3+^ in the presence of hydrogen peroxide (H_2_O_2_) [1]. The produced Mn^3+^ is stabilized by organic acid and forms a Mn^3+^-organic acid complex that acts as a diffusible oxidizer, which in turn attacks a variety of aromatic compounds, including lignin, industrial dyes, and other organic contaminants [2, 3]. Crystal structures and spectroscopic studies demonstrated that MnP is a two-domain, globular protein that consists of 11 or 12 α-helices. A heme prosthetic group, most commonly Fe protoporphyrin IX, is located between the two domains [4]. Two Ca^2+^-binding sites, which provide structural support, are located on both sides of the heme group, and a Mn-binding site, which is required for catalysis, is located near the internal heme propionate [4, 5]. In addition, MnP has four or five disulfide bridges that stabilize its protein structure [6].

Commercial applications of fungal MnP are mainly based on the high level of protein expression of some fungal strains. However, as described by Saroj et al. [7], the yield of native MnP in its native hosts was too low, and approximately 1.0 to 5 mg of purified recombinant MnP was obtained per liter of extracellular culture fluid. And an appropriate medium composition and critical culture conditions are necessary to induce fungi to produce ligninolytic enzymes [8]. Therefore, improving the enzyme yield and reducing the cost of preparation are needed to meet the requirements of commercial use. In recent years, the production of recombinant proteins has been among the main options. Both homologous and heterologous expression in prokaryotic and eukaryotic systems have been used to achieve the overproduction of MnP. For instance, homologous expression systems in *Phanerochaete chrysosporium* [9] and *Pleurotus ostreatus* [10] used a primary metabolic promoter, but the resulting MnP yield was still low. *Pichia pastoris*, which is a common heterologous expression system, has been used to produce active MnP at a maximum yield of only 120 U/L [11]. In addition, numerous studies have used *Escherichia coli* to express MnP [12, 13]. In general, the recombinant protein accumulated in the form of in inclusion bodies is an inactive, insoluble form; functional, active MnP could be obtained by in vitro refolding based on the incorporation of exogenous hemin and Ca^2+^. Nevertheless, an efficient process to produce high yields of the recombinant enzyme is still lacking, even after optimizing the refolding procedure [12, 14]. Additionally, refolding is a result of trial and error, and it requires much effort to obtain a renatured protein. Therefore, it is of great interest and importance to explore soluble protein production in *E. coli*. In this regard, alternative methods have been examined, such as choosing a suitable vector and host [15], changing the type of promoter [16], switching the location of an affinity tag [16], decreasing the growth temperature [17], optimizing codon usage [18], adding osmolytes to the expression medium [19], coexpressing chaperones [20], and site-directed mutagenesis [21]. However, thus far, no strategy has yielded soluble MnP in *E. coli*.

*Irpex lacteus* F17 is a local, MnP-producing, white-rot basidiomycete fungus, and an MnP-encoding gene, *imnp*, from this species has been cloned and expressed in *E. coli* by our laboratory [22]. As expected, the expressed protein accumulated in an insoluble form as inclusion bodies. Subsequent in vitro refolding and incorporating exogenous hemin into the recombinant protein led to the formation of biologically active MnP [22]. In the present study, we further optimized the in vitro refolding conditions to improve the efficiency of renaturation from inclusion bodies using a one-factor-at-a-time method and an orthogonal experimental design based on our previous results. In parallel, we used a strategy to express MnP in a soluble form in *E. coli* by adding chemicals to the cultures, and simultaneously lowering the growth temperature during protein expression. Additionally, the biochemical and spectroscopic characterizations of soluble and refolded MnP were analyzed, and their abilities to decolorize azo dyes were investigated.

## Results

### Optimal conditions for refolding MnP from inclusion bodies

In the single-factor experiment, the refolding efficiency was estimated from different shades of red in a 48 deep-well plate by changing various refolding parameters, as well as their concentrations or quantities, to preliminarily screen for the important factor(s). The results indicated that urea, GSSG, glycerol, and pH significantly affected the enzyme activity, while MnSO_4_ and KCl had no obvious effects. No activity was detected if CaCl_2_ and hemin were omitted from the refolding solution. To search for the optimal combination of significant factors, as well as their concentrations or quantities, an orthogonal experiment design, which consisted of six factors (urea, CaCl_2,_ hemin, glycerol, GSSG, and pH), was tested at three levels using the L_18_(3^6^) orthogonal array. Details of the three levels for each factor were shown in Table 1. Eighteen experiments according to the L_18_(3^6^) orthogonal array were performed. Results of the effects of the six factors on enzymatic specific activity were presented in Table 2. K_1_ was the sum of the specific activities for each factor when the factor level is 1, and k_1_ was the average number of K_1_, and so on, for K_2_ and K_3_, as well as for k_2_ and k_3_. Meanwhile, R was the difference of the largest and smallest k values of each factor. A large value of R indicates that the effect of factor on the results is significant. As shown in Table 2, assay “6” produced the maximum MnP specific activity of 3.63 U/mg, which was almost twofold higher than that of the optimized condition obtained in the single-factor experiment (1.88 U/mg). In addition, according to the maximum of k_1_, k_2_ and k_3_ of each factor, a combination (i.e., 1.0 M urea, 150 mM CaCl_2_, 10 μM hemin, 10% glycerol, 0.5 mM GSSG, and pH 8.5) was further tested, and the specific activity of refolded MnP was 2.65 U/mg, indicating that it was inferior to experiment “6”. Thus, the optimized refolding solution comprised 1.5 M urea, 150 mM CaCl_2_, 25 μM hemin, 10% glycerol, 0.5 mM GSSG, 0.05 mM MnSO_4_, and 20 mM KCl in 50 mM Tris-HCl (pH 8.5) ﻿at 4 °C for 24 h.﻿ The value of R in Table 2 showed that pH and GSSG had important effects on specific activity of refolding MnP.

**Table 1:** Contents of orthogonal factors and levels L_18_(3^6^)

Factor level	A Urea (M)	B CaCl_2_ (mM)	C Hemin (μM)	D Glycerol (%)	E GSSG (mM)	F pH
1	1.0	75	5.0	10	0.5	8.0
2	1.5	100	10	15	0.7	8.5
3	2.0	150	25	20	1.0	9.0

**Table 2:** Effect of six factors on specific activity of refolded MnP

Experiment	A Urea (M)	B CaCl_2_ (mM)	C Hemin (μM)	D Glycerol (%)	E GSSG (mM)	F pH	Specific activity (U/mg)
1	1	1	1	1	1	1	1.63 ± 0.23
2	1	2	2	2	2	2	1.89 ± 0.59
3	1	3	3	3	3	3	0.65 ± 0.02
4	2	1	1	2	2	3	0.86 ± 0.15
5	2	2	2	3	3	1	0.86 ± 0.04
6	2	3	3	1	1	2	3.63 ± 0.19
7	3	1	2	1	3	2	2.04 ± 0.24
8	3	2	3	2	1	3	1.10 ± 0.06
9	3	3	1	3	2	1	1.01 ± 0.25
10	1	1	3	3	2	2	2.23 ± 0.39
11	1	2	1	1	3	3	1.83 ± 0.25
12	1	3	2	2	1	1	3.38 ± 0.06
13	2	1	2	3	1	3	1.02 ± 0.23
14	2	2	3	1	2	1	0.86 ± 0.07
15	2	3	1	2	3	2	2.03 ± 0.10
16	3	1	3	2	3	1	0.96 ± 0.12
17	3	2	1	3	1	2	1.52 ± 0.36
18	3	3	2	1	2	3	1.16 ± 0.09
K_1_	11.61	8.742	8.88	11.148	12.282	8.7	
K_2_	9.258	8.058	10.35	10.218	8.01	13.338	
K_3_	7.788	11.862	9.432	7.29	8.37	6.618	
k_1_	1.935	1.457	1.48	1.858	2.047	1.45	
k_2_	1.543	1.343	1.725	1.703	1.335	2.223	
k_3_	1.298	1.977	1.572	1.215	1.395	1.103	
R	0.637	0.634	0.245	0.643	0.712	1.12	

Specific activity was obtained from the orthogonal design experiment. Each value is the average of three independent runs of experiment

K_1_ represents the sum of the specific activity of each factor if the factor level is 1, K_2_ represents the sum of the specific activity of each factor if the factor level is 2, and so K_3_. k_1_ represents the average number of K_1_, and so on, for k_2_ and k_3_. R represents the difference of the largest and smallest k values of each factor

### Optimal conditions for expressing soluble MnP

Similarly, the procedure searching for the optimal expression conditions for soluble MnP was similar to that used to optimize MnP refolding from inclusion bodies. According to the results of a single-factor experiment, Triton X-100 played a significant role in the expression of soluble MnP. Tween-80, glycerol, and ethanol improved the activity of soluble MnP, while sorbitol, L-proline, and glycine had no distinct impact on the expression of soluble MnP. Thus, an orthogonal experimental design comprising four factors (Triton X-100, Tween-80, glycerol, and ethanol), with each factor being tested at three levels, was performed using the L_9_(3^4^) orthogonal array (Table 3). Results of the effects of the four factors on enzymatic specific activity were presented in Table 4. As shown, assay “1” produced a specific activity (0.411 U/mg), which was higher than that of the optimized condition obtained in the single-factor experiment (0.391 U/mg). In addition, according to the maximum of k_1_, k_2_ and k_3_ of each factor in Table 4, a combination was further tested, which resulted in a maximum of 0.56 U/mg, higher than the specific activity of experiment “1”. As a result, the production of soluble MnP could be induced by the addition of 0.3 mM IPTG, along with 0.25% Triton X-100, 0.25% Tween-80, 0.5% glycerol, and 1% ethanol, to the cell cultures, followed by cultivation for 16 h at 16 °C. The value of R in Table 4 indicated that Triton X-100 was the most important factor for expressing soluble MnP in *E. coli*.

**Table 3:** Contents of orthogonal factors and levels L_9_(3^4^)

Factor level	A	B	C	D
	Triton X-100	Tween-80	Glycerol	Ethanol
	(%)	(%)	(%)	(%)
1	0.25	0.25	0.25	1.0
2	0.5	0.5	0.5	1.5
3	1.0	1.0	1.0	2.0

**Table 4:** Effect of four factors on specific activity of soluble MnP

Experiment	A	B	C	D	Specific activity
	Triton X-100	Tween-80	Glycerol	Ethanol	(U/mg)
	(%)	(%)	(%)	(%)	
1	1	1	1	1	0.411 ± 0.03
2	1	2	2	2	0.355 ± 0.02
3	1	3	3	3	0.361 ± 0.14
4	2	1	2	3	0.320 ± 0.03
5	2	2	3	1	0.185 ± 0.01
6	2	3	1	2	0.208 ± 0.07
7	3	1	3	2	0.167 ± 0.02
8	3	2	1	3	0.121 ± 0.03
9	3	3	2	1	0.252 ± 0.27
K_1_	1.127	0.897	0.741	0.849	
K_2_	0.714	0.66	0.927	0.729	
K_3_	0.54	0.822	0.714	0.801	
k_1_	0.376	0.299	0.247	0.283	
k_2_	0.238	0.22	0.309	0.243	
k_3_	0.18	0.274	0.238	0.267	
R	0.196	0.079	0.071	0.04	

Specific activity was obtained from the orthogonal design experiment. Each value is the average of three independent runs of experiment

K_1_ represents the sum of the specific activity of each factor if the factor level is 1. K_2_ represents the sum of the specific activity of each factor if the factor level is 2, and so K_3_. k_1_ represents the average number of K_1_, and so on, for k_2_ and k_3_. R represents the difference of the largest and smallest k values of each factor

### Purification of soluble and refolded MnP

**Fig. 1:**
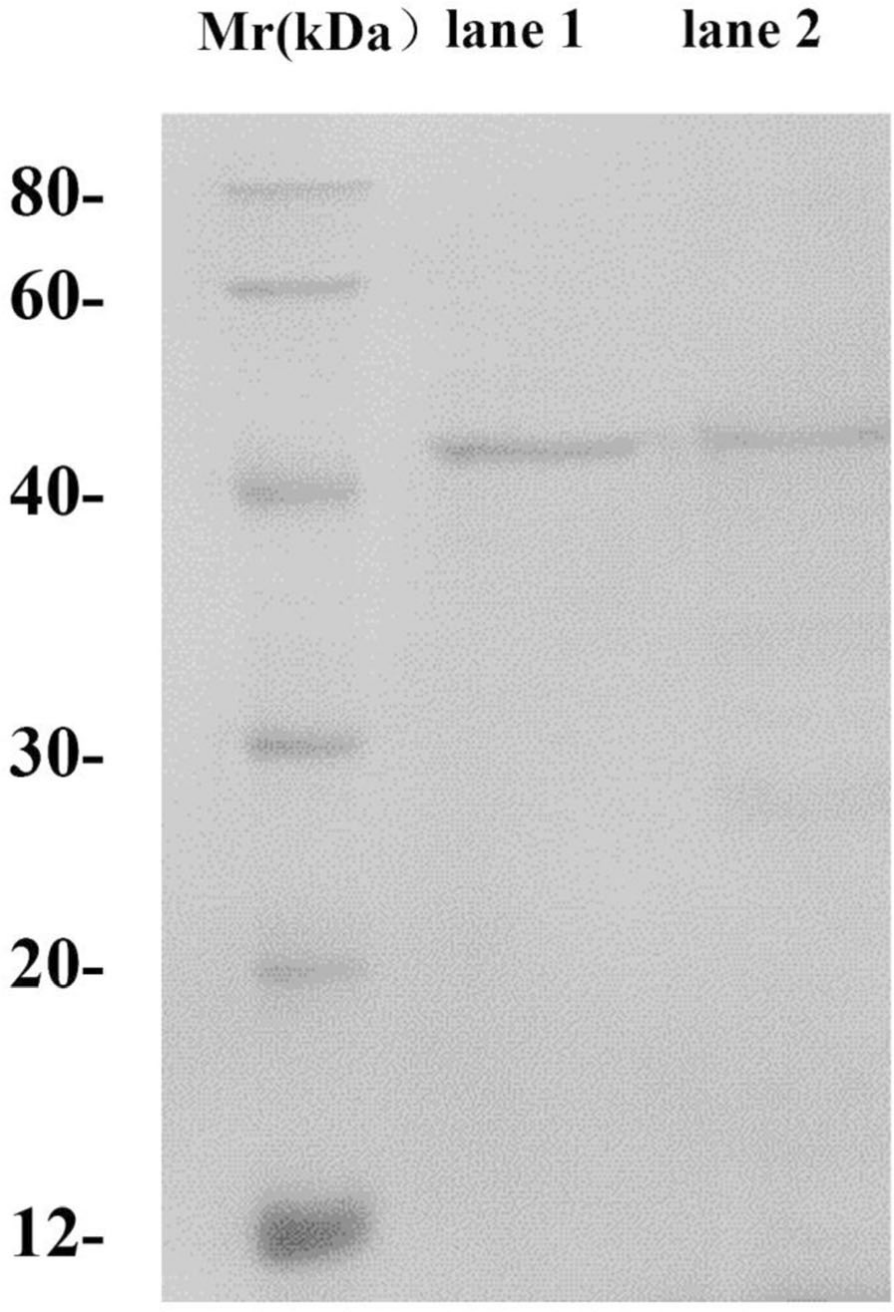
SDS-PAGE of recombinant MnP purified by a Ni^2+^ His-tag column. Lane M: protein standards; Lane 1: purified refolded MnP; Lane 2: purified soluble MnP

Two forms of the recombinant MnP were obtained from *E. coli* under the optimal conditions for insoluble inclusion body and for soluble protein, respectively. Both soluble and refolded MnP, each with a C-terminal, six-histidine tag, were purified by Ni-NTA affinity chromatography. They were visualized as a single band on SDS-PAGE, and they exhibited a similar molecular weight of 43 kDa (Fig. 1). Under the optimal refolding conditions, the refolding yield of dialyzed MnP obtained from inclusion bodies was 13%, and the yield of purified MnP was 2.4% (Table 5). The final recovery, which was calculated from the total activity, was approximately 78%. For soluble MnP, approximately 1.5 mg of purified, active MnP was obtained from 2 L of *E. coli* fermentation broth.

**Table 5:** Isolation and purification of MnP from inclusion bodies

Sample	Protein concentration (mg/ml)	Protein (mg)	Specific activity (U/mg)	Total activity (U)	Yield (%)
Inclusion body	18.7	93.5			
Dialyzed	0.43	12.35	3.04	37.5	100
Ni-NTA	0.076	2.26	12.9	29.15	78

### Biochemical characterization of soluble and refolded MnP

#### Enzyme kinetic properties

Table 6 shows the kinetic constants of soluble and refolded MnP using Mn^2+^ and H_2_O_2_ as substrates. The *K*_*m*_ (336 μM) of soluble MnP for Mn^2+^ was higher than that (148 μM) of refolded MnP, and the *K*_*m*_ (23.5 μM) of soluble MnP for H_2_O_2_ was reduced 90% than that (234.5 μM) of refolded MnP. The *k*_*cat*_ */K*_*m*_ values of refolded MnP for Mn^2+^ and H_2_O_2_ were 12.2-fold and 2.9-fold higher than those of soluble MnP, respectively. However, the *k*_*cat*_ */K*_*m*_ values of the two forms of recombinant MnP were both lower than that of the native MnP for Mn^2+^ (9.62 × 10^3^ (mmol)^−1^ L s^−1^) and H_2_O_2_ (5.22× 10^4^ (mmol)^−1^ L s^−1^), respectively.

**Table 6:** Kinetic parameters of refolded and soluble MnP

Enzyme	*Km* (Mn^2+^) μmol L^−1^	*Vmax* (Mn^2+^) μmol L^−1^ min^−1^	*Kcat* (Mn^2+^) s^−1^	*Kcat/Km* (Mn^2+^) (mmol)^−1^ L s^−1^
Refolded MnP	148 ± 7.1	1276 ± 23.3	141.9 ± 2.62	930 ± 30
Soluble MnP	336 ± 8.0	236.6 ± 24.7	25.6 ± 2.7	76 ± 9.9
Native MnP	100.7 ± 0.87	1116.3 ± 18.3	969 ± 22.6	9620 ± 420
Enzyme	*Km* (H_2_O_2_) μmol L^−1^	*Vmax* (H_2_O_2_) μmol L^−1^ min^−1^	*Kcat* (H_2_O_2_) s^−1^	*Kcat/Km* (H_2_O_2_) (mmol)^−1^ L s^−1^
Refolded MnP	234.5 ± 12	3326.5 ± 179	554.4 ± 29	2370 ± 7
Soluble MnP	23.5 ± 4.9	308.1 ± 26.9	19 ± 1.7	820 ± 100
Native MnP	30.7 ± 4.4	1844.5 ± 25	1601.1 ± 34.8	52200 ± 450

#### Temperature stability and pH optimum

**Fig. 2:**
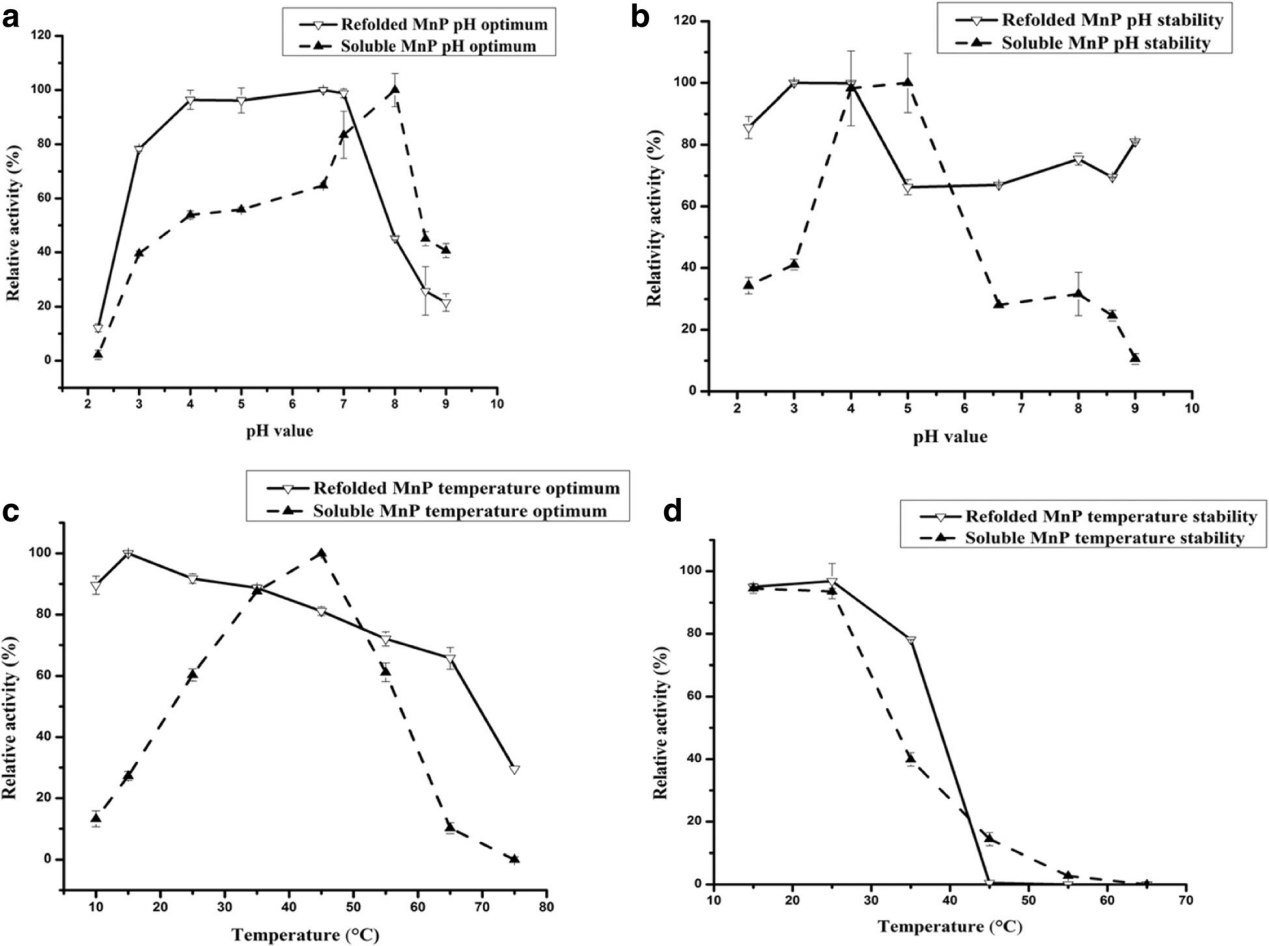
Effect of pH and temperature on the activity and stability of refolded and soluble MnP. And MnP activity at the beginning was considered to be 100%. Deviation values are standard deviations based on triplicate determinations. **a** The pH optimum of refolded and soluble MnP. The MnP activity was determined in the citrate-phosphate buffer, pH 2.2-8.0 and Tris–HCl, pH 8.6-9.0, at 25 °C. **b** The pH stability of refolded and soluble MnP. Refolded and soluble MnP was incubated for 6 h at 25 °C in various pH vaules in citrate-phosphate (2.2-8.0) or Tris–HCl buffer (8.6-9.0). The residual MnP activity was measured according to the method of MnP activity assay. **c** The temperature optimum of refolded and soluble MnP. The enzyme reaction was performed in 0.11 M sodium lactate buffer, pH 4.5 at 10–75 °C. **d** The temperature stability of refolded and soluble MnP. Refolded and soluble MnP were incubated for 6 h at 15–65 °C. The residual MnP activity was measured according to the method of MnP activity assay

As illustrated in Fig. 2a, soluble MnP showed less than 65% relative activity (the ratio of actual to maximum activity) in a broad pH range (2.2–6.6). However, a rapid increase in activity occurred at pHs higher than 7.0, and the optimal pH was 8.0. Additionally, the enzyme was optimally stable at pH 5.0 during a 6-h incubation in citrate-phosphate buffer (pH 5.0). Unlike soluble MnP, refolded MnP showed maximum activity at pH 6.5, and retained more than 80% of its activity over a wide range of pH (3.0–7.0). In addition, the pH stability of refolded MnP was superior to that of soluble MnP (Fig. 2b). As shown in Fig. 2c and d, soluble MnP exhibited the highest activity at 45 °C, and retained 90% of its activity after a 6-h incubation at temperatures below 25 °C. Refolded MnP displayed optimum activity at 15 °C and was very stable below 35 °C; no activity was detected at 45 °C after incubation for 6 h.

#### Substrate specificity

To examine the substrate specificity of soluble and refolded MnP, the monocyclic phenolic compounds DMP and guaiacol, as well as the polycyclic compound ABTS, were selected as substrates. The results showed that the oxidation of these substrates by soluble MnP was similar to that for refolded MnP, and they were all dependent upon Mn^2+^ (Table 7). The results indicated that both of the soluble and refolded MnP are powerful oxidants that are capable of oxidizing aromatic compounds.

**Table 7:** Substrate specificities of refolded and soluble MnP

Substrate	Concentration (mM)	Emax (M^−1^ cm^−1^)	Wavelength (nm)	Refolded MnP (U L^−1^)		Soluble MnP (U L^−1^)	
				Mn^2+^ present	Mn^2+^ absent	Mn^2+^ present	Mn^2+^ absent
ABTS	1	36000	420	344.93 ± 0.03	37.73 ± 0.01	310.0 ± 0.046	24.4 ± 0.006
DMP	1	49600	469	105.42 ± 0.04	0.32 ± 0.001	104.35 ± 0.05	0
Guaiacol	1	12100	456	122.31 ± 0.003	2.27 ± 0.001	112.40 ± 0.008	0

### Decolorization of Acid Red 18 and Orange G

**Fig. 3:**
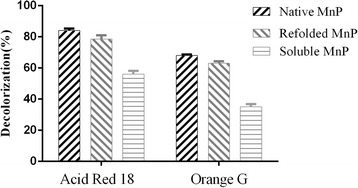
Acid Red 18 and Orange G decolorization by refolded and soluble MnP

Acid Red 18 and Orange G, as important azo dyes, have been extensively used in the textile, printing, food, and drug industries, and they are usually released into industrial wastewater. Accordingly, we chose them to examine the degradation capability of soluble and refolded MnP. As shown in Fig. 3, the decolorization efficiency of Acid Red 18 was 63 ± 1.41% for the soluble MnP after a 60-min reaction at pH 4.0 and 35 °C, while the decolorization efficiency was 78.5 ± 0.71% for the refolded MnP. Moreover, for Orange G, 35 ± 0.01 and 56 ± 0.03% decolorization efficiencies were observed for soluble and refolded MnP, respectively. However, the decolorization efficiencies of the two forms of recombinant MnP were both lower than that of the native MnP for Acid Red 18 (84 ± 1.3%) and Orange G (68 ± 0.6%), respectively.

### Spectroscopic characterization of soluble and refolded MnP

#### CD spectra and UV-VIS spectra

**Fig. 4:**
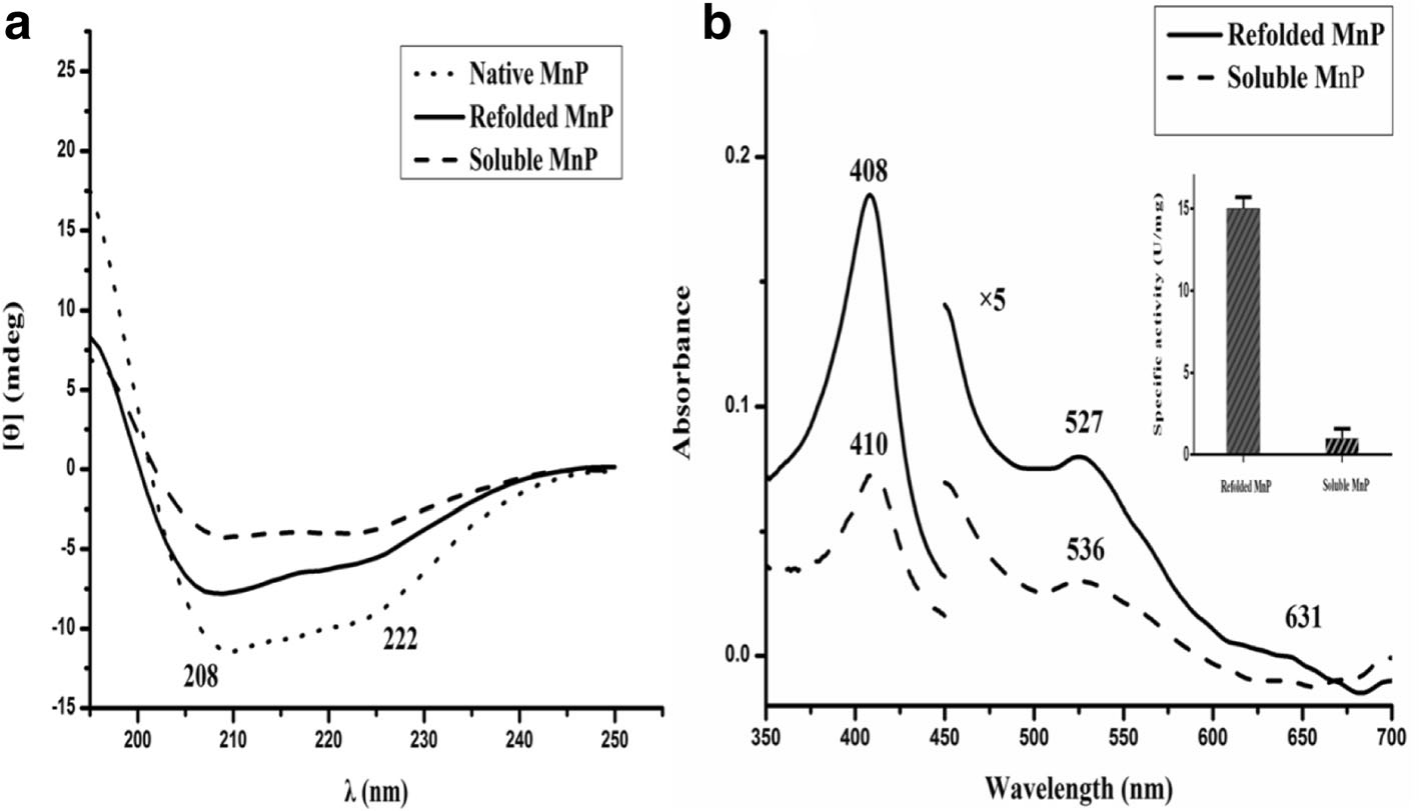
The far-UV CD and absorbance spectra of refolded and soluble MnP. **a** The far UV-CD circular dichroism spectra of native, refolded and soluble MnP at room temperature and 0.1 mg/ml of protein concentration in 0.15 M phosphate buffer at pH 6.5. **b** Electronic absorption spectra of refolded and soluble MnP at room temperature. The region between 450 and 700 nm has been expanded (×5 absorbance). Enzymatic specific activities are represented in the inset

It is well known that a circular dichroism (CD) spectrum in far-UV region provides information on the secondary structure of a protein. The far-UV CD spectra of soluble and refolded MnP exhibited two negative ellipticity bands at 208 nm and 222 nm (Fig. 4a), respectively, displaying characteristics of an α-helical structure, but they showed notable differences. Furthermore, the α-helix and the β-sheet contents of the two MnPs were calculated using the SELCON3 program, and the data showed that the helical content of soluble MnP (18.7%) was lower than that of refolded MnP (24.3%), whereas the β-sheet content of soluble MnP (27%) was higher than that of refolded MnP (18.2%).

To evaluate the secondary structure of soluble and refolded MnP, we investigated the far-UV CD spectrum of the purified, native MnP from fungus *Irpex lacteus* F17 using CD spectrometer under the same conditions, as shown in Fig. 4a. The native fungal MnP was obtained according to our previous experimental protocols described in the literature [23]. On the basis of the analysis of SELCON3 program, 39.7% α-helix and 13.7% β-sheet were involved in the structure of CD of native MnP produced by fungus *Irpex lacteus* F17.

The UV-VIS spectrum of soluble MnP was similar to that of refolded MnP (Fig. 4b); however, their absorption intensities obviously differed. Refolded MnP exhibited a Soret peak at 408 nm, Q band at 527 nm and a charge transfer (CT) band at 631 nm, while soluble MnP shifted the Soret peak to 410 nm, Q band to 536 nm with a tiny CT band at 631 nm, and the absorbance of soluble MnP was clearly lower than that of refolded MnP. The Reinheitszahl (R_Z_) values (calculated from the A408/A280 and A410/A280 absorption ratios) for the purified, refolded MnP and soluble MnP were 2.5 and 1.1, respectively, indicating that the heme environment of the two enzymes differed. These results are in agreement with the lower specific activity of soluble MnP compared with refolded MnP, as shown in the inset of Fig. 4b.

#### Fluorescence spectra

**Fig. 5:**
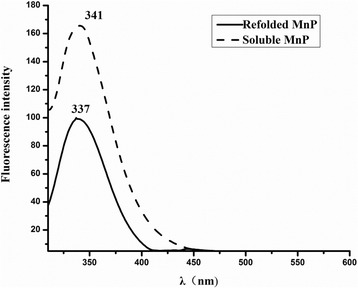
The emission fluorescence spectra of refolded and soluble MnP. The emission fluorescence spectra of refolded and soluble MnP at room temperature and 0.1 mg/ml of protein concentration in the phosphate buffer at pH 6.5

Figure 5 showed the fluorescence spectra of soluble and refolded MnP at room temperature following excitation at 295 nm. As shown, refolded MnP had a maximum emission at 337 nm, whereas the maximum emission of soluble MnP occurred at 341 nm, which represented a 4-nm red shift and an increased quantum yield.

#### Raman spectra

**Fig. 6:**
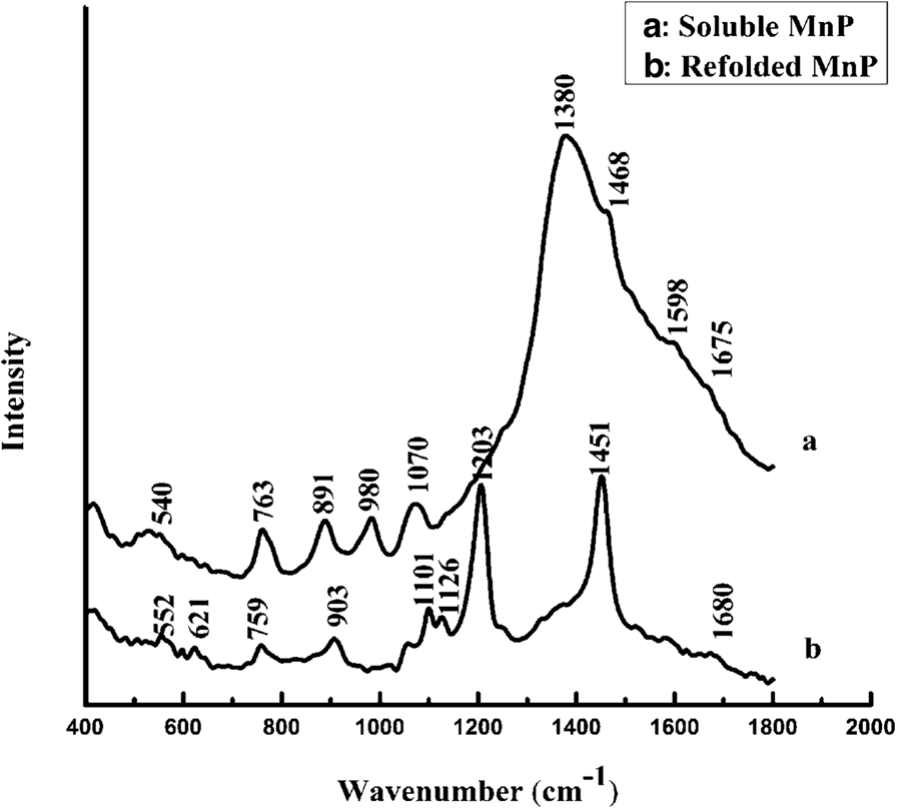
The Raman spectra of refolded and soluble MnP

Figure 6 represented the Raman spectra of soluble and refolded MnP at room temperature following excitation at 785 nm. The C-C stretching vibration near 890–945 cm^−1^ of Raman spectra indicated that soluble and refolded MnP were primarily α-helix structures. The relative weak amide I bands centered at 1598 cm^−1^ and 1675 cm^−1^ were both correlated with an increase in β-sheet contents for soluble MnP, while the β-sheet structure of refolded MnP was involved in an amide I band centered at 1680 cm^−1^. The band at 1070 cm^−1^ exhibited in soluble MnP and the bands at 1101 and 1126 cm^−1^ presented in refolded MnP, originated from C-N stretching vibrations, revealed some conformation modifications between the two enzymes in the protein polypeptide backbone. A low intensity band at 540 cm^−1^ appeared in the spectrum of soluble MnP indicated the existence of the C-C-S-S-C-C stretching vibrations in trans-gauche-trans arrangements [24], whereas the structure of disulfide bonds was revealed by a weak band near 552 cm^−1^ in refolded MnP. Furthermore, a band at 759 cm^−1^ indicated the existence of tryptophan in refolded MnP, while a change occurred in soluble MnP by shifting the band to 763 cm^−1^. Also, the phenylalanine bands of refolded MnP were observed at 621 cm^−1^ and 1203 cm^−1^. Additionally, the bands at 1380 cm^−1^ and 1468 cm^−1^ for soluble MnP, and a band at 1451 cm^−1^ for refolded MnP were attributed to the CH_2_ bending vibrations, showing some changes in protein side chains between the two enzymes.

#### Size exclusion chromatography and dynamic light scattering analysis

**Fig. 7:**
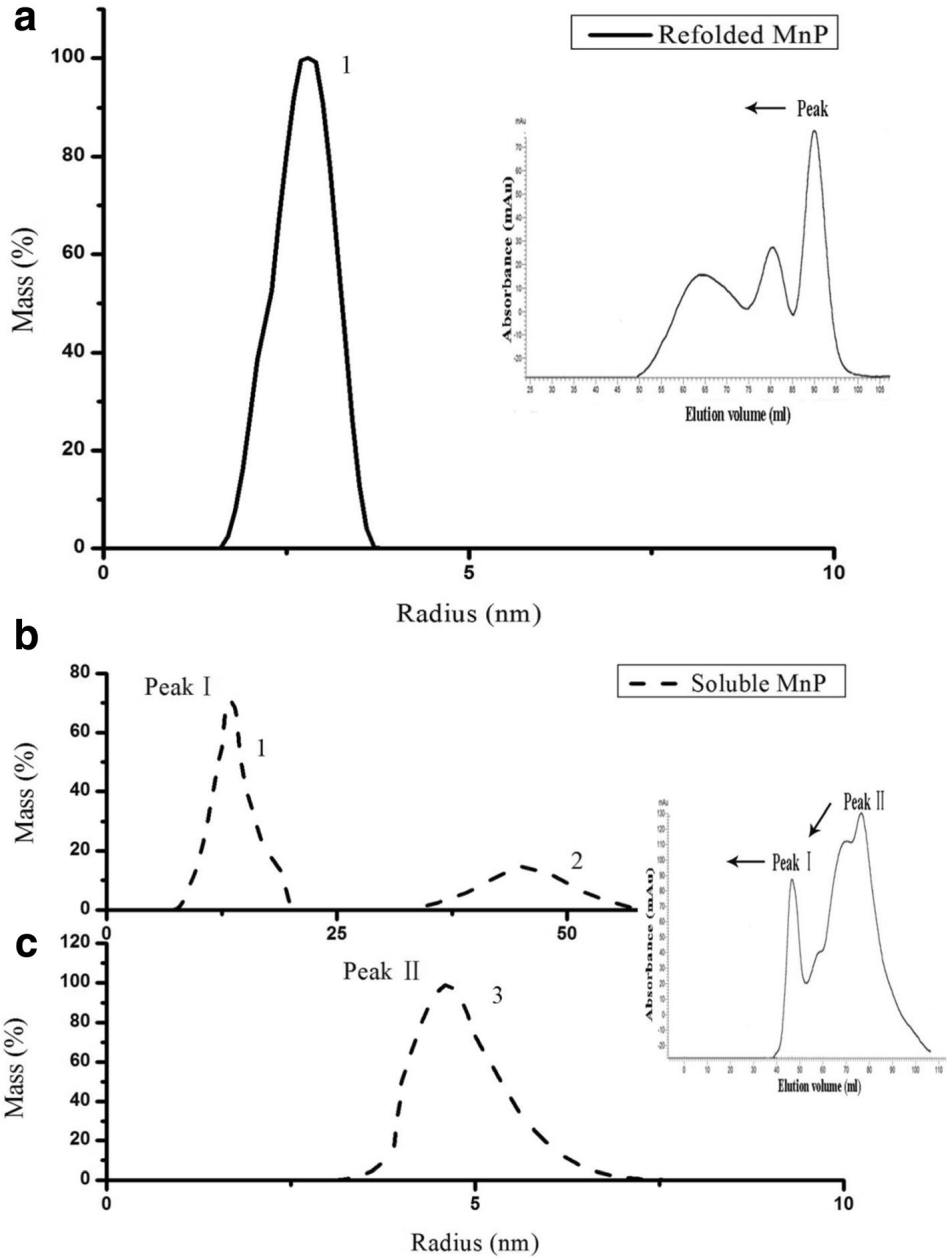
Protein size analysis of refolded and soluble MnP. **a** Dynamic light scattering results of refolded MnP. The percentage of the protein mass (%) was plotted against the hydrodynamic radius (nm). Size exclusion chromatography of refolded MnP purified from Ni-NTA affinity chromatography was shown in the inset. Protein samples were collected after elution with 120 and 200 mM imidazole using 5-ml Ni Sepharose™ 6 Fast Flow. Elution profile of protein was plotted against the absorbance at 280 nm. **b** & **c** Dynamic light scattering results of soluble MnP. The percentage of the protein mass (%) was plotted against the hydrodynamic radius (nm). Size exclusion chromatography of soluble MnP from Ni-NTA affinity chromatography was shown in the inset

To further investigate the structural properties of soluble and refolded MnP, SEC and DLS experiments were used to analyze their protein behaviors and status in solution. The DLS distribution patterns of soluble and refolded MnP were given in Fig. 7 and the corresponding elution profiles were shown in the inset. The data from SEC and DLS were summarized in Table 8. The results revealed some notable differences between the two enzymes. In the case of refolded MnP (Fig. 7a, Table 8), the main peak in FPLC column showed a high specific activity (15.0 U/mg) and a high Rz (3.1), which corresponded to a monomodal distribution in DLS pattern (Fig. 7a). The hydrodynamic radius of the particle 1 which constituted 99.6% of the protein was 2.9 ± 0.5 nm, and represented an estimated molecular weight of 42.0 kDa. The data is corresponding with the determined MW (43.0 kDa) of recombinant MnP on SDS-PAGE, as shown in Fig. 1, and is also consistent with that of native MnP from *Irpex lacteus* F17, as reported in the literature [23], as well as the molecular range of the MnP family (38–50 kDa) [25, 26]. Thus, it could be concluded that refolded MnP was a monomer in solution. In the case of soluble MnP (Fig. 7, Table 8), two prominent peaks of I and II in FPLC column showed low MnP activities and low Rz values, which corresponded to peak I and peak II of soluble MnP in DLS pattern, respectively (Fig. 7b, c). The DLS results showed that the size distribution of soluble MnP peak I displayed a bimodal distribution with the mass percentage of 70.9% (particle 1) and 22.3% (particle 2), respectively (Fig. 7b); particle 1 centered at around 12.9 nm was dominant and indicated low aggregated oligomers, while particle 2 around 45.3 nm was comparatively weak and showed large aggregated status. Meanwhile, DLS of soluble MnP also showed a peak II with an average hydrodynamic radius of 4.5 nm (particle 3), which constituteed 99.3% of the protein and represented an estimated molecular weight of 111.0 kDa (Fig. 7c). This particle 3 probably was related to the formation of protein dimers. Therefore, it could be suggested that the overwhelming majority of soluble MnP was the oligomeric status in solution under the experimental conditions, different from a single monomeric form of refolded MnP.

**Table 8:** Summary of the data from DLS and FPLC for refolded and soluble MnP

Size distribution	Radius (nm)	Molecular weight (kDa)	Mass (%)	Specific activity (U/mg)	Rz (Soret/280)
refolded MnP					
Particle 1	2.9 ± 0.5	42.0	99.6	15.0 ± 2.1	3.1 ± 0.6
soluble MnP peak I					
Particle 1	12.9 ± 0.7	1314.0	70.9	0.6 ± 1.5	1.0 ± 0.3
Particle 2	45.3 ± 1.4	25199.0	22.3		
soluble MnP peak II					
Particle 3	4.5 ± 0.8	111.0	99.3	0.2 ± 0.8	0.3 ± 1.1

“Particle 1” of refolded MnP was presented in Fig. 7a; “Particle 1”, “Particle 2” and “Particle 3” of soluble MnP were presented in Fig. 7b and Fig. 7c. The results of hydrodynamic radius, specific activity and Rz were given as the mean ± standard deviation, calculated from at least three repetitions

## Discussion

Our previous studies indicated that the MnP preprotein from *I. lacteus* F17 is composed of 359 amino acid residues, including a 26-amino acid signal peptide and four disulfide bridges essential for the native conformation of the enzyme [22]. A cDNA encoding the mature 333-amino acid MnP protein without the signal sequence was subcloned into pET28a and expressed in *E. coli* Rosetta (DE3) cells. As expected, this protein formed inclusion bodies and required in vitro refolding to convert it into an active enzyme*,* as is true for recombinant MnP from *P. chrysosporium* [12] and *Phlebia radiata* [27].

One of the purposes of the present study was to enhance the MnP yield by optimizing the in vitro refolding conditions. Orthogonal design is a good mathematical method used for planning multivariate tests [28]. Its greatest applications have been in healthcare evaluations or comparative effectiveness research [29]. Using an orthogonal design in the present study, the optimal refolding conditions were obtained, and the MnP yield attained was higher than that obtained in our previous studies, even surpassing those reported for recombinant MnP from *P. chrysosporium* [12], horseradish peroxidase [30], and lignin peroxidase [31].

Another purpose of the study was to determine whether MnP could be expressed in a soluble form in *E. coli* to avoid the tedious process of renaturing inclusion bodies. In this study, a biologically active form of soluble MnP was expressed by simultaneously decreasing the expression temperature and adding chemicals to the culture medium. Namely, the recombinant MnP could be expressed as a soluble protein in the presence of four chemicals in the medium, at 16 °C. At low temperature, the rate of protein synthesis slows; thus, proteins have sufficient time to fold, which increases their solubility [32]. Esposito et al. [33] also reported that a lower expression temperature improved the solubility of *E. coli*-expressed proteins, while a higher expression temperature was conducive to the production of insoluble protein in *E. coli* Rosetta-gami strains [34]. Our results also demonstrated that an expression temperature of 16 °C could increase the production of soluble MnP, but an expression temperature of 37 °C could not. In addition, some chemical additives play a useful role in increasing the solubility of recombinant proteins in *E. coli* [19]. Triton X-100 and Tween-80 are surfactants that can change the osmotic pressure in bacterial membranes [35]. Moreover, Triton X-100 supplementation in culture medium can hinder the formation of inclusion bodies in *E. coli*, and facilitate the secretion of recombinant proteins to the periplasmic space or culture medium [36]. Glycerol is commonly used as a protective agent to increase protein stability, as it is able to decrease the surface tension of water and increase viscosity [37]. An optimum glycerol concentration can stabilize protein structures, even at high protein concentrations. Ethanol has also been shown to increase the fraction of soluble protein [38]. Steczko et al. [39] reported that adding 3% ethanol dramatically increased the amount of soluble, active soybean seed lipoxygenase L-1. Using an orthogonal design, we determined the optimized combination of Triton X-100, Tween-80, glycerol, and ethanol, which resulted in the expression of soluble MnP in *E. coli* Rosetta (DE3) cells. Thus, it was also confirmed that these solutes did not impair cell metabolism and that they were compatible solutes [40]. MnP activity was detected in the cell lysates after hemin and Ca^2+^ supplementation at pH 8.0. However, recombinant MnP was not secreted into the culture medium, as determined by SDS-PAGE and enzyme activity assays, in our study.

As a result, soluble MnP was successfully induced and expressed in *E. coli* when grown in the presence of the four additives at 16 °C, as opposed to forming inclusion bodies. These additives could be used as agents to increase the yield of soluble proteins at lower temperatures. Although the concrete mechanism underlying this phenomenon is unclear, a case like this has been observed previously. Bacterial cytoplasm is a rather reducing environment, it generally inhibits the formation of disulfide bonds [41], while the periplasmic space provides an oxidative environment that facilitates proper protein folding [42]. Lee and Choi [36] described the secretory and extracellular production of recombinant proteins, which were exported to periplasm space or culture medium, using protein secretion systems in *E. coli*. Prasad et al. [19] suggested that chemical chaperones, such as sorbitol and arginine, increased the solubility of recombinant proteins. Yang et al. [43] reported that culturing conditions had effects on the production of heterologous proteins in *E. coli*. However, in the present study, the detailed information to reveal which pathways take part in the formation of soluble MnP have not been determined and need to be further investigated.

It is worth mentioning that hemin is crucial to activate soluble MnP. Heme is a non-covalently bound prosthetic group in MnP; hence, adding exogenous hemin to the cell lysates is necessary to yield an active holoenzyme. Additionally, Ca^2+^ supplementation is crucial to achieve the correct conformation, as well as the stability, of recombinant MnP [44, 45], because both Ca^2+^ ions are easily lost in a thermal or alkaline environment [6, 46].

Compared with refolded MnP, soluble MnP showed low catalytic efficiencies (*k*_*cat*_ */K*_*m*_) for Mn^2+^ and H_2_O_2_ substrates and was more susceptible to H_2_O_2_. The kinetic parameters are in agreement with those obtained from the spectroscopic characterizations. Additionally, both of the soluble and refolded MnP exhibited a broad range of substrate specificities in the presence of Mn^2+^. Interestingly, soluble MnP had an optimum pH of 8.0 and an optimum temperature of 45 °C, which are obviously different from those of refolded MnP (pH 6.5 and 15 °C, respectively) and most of the reported fungal MnPs [47–49]. Furthermore, the two enzymes had the ability to decolorize the azo dyes Acid Red 18 and Orange G, but the decolorization efficiency of soluble MnP was lower than that of refolded MnP.

Experiments of CD and UV-VIS absorption spectra showed certain differences in the secondary structures, Soret peaks and other bands between soluble and refolded MnP (Fig. 4a and b). Compared with refolded MnP, soluble MnP showed the Soret peak height reduced by about 40% and red-shifted 2 nm, Q band reduced by about 37% and red-shifted 9 nm, as well as an inconspicuous CT band (631 nm). A characteristic CT band at 631 nm was observed in the spectrum of refolded MnP, indicating a high-spin configuration of the protein, because the wavelength of the region between 600 and 650 nm is sensitive to changes in the heme microenvironment [50, 51]. This band, assigned to a charge transfer transition from the porphyrin to the iron, was typical of a high-spin heme Fe^3+^ states [51]. The specific activities and R_Z_ values demonstrated that soluble MnP was less active than refolded MnP. The CD spectra analyses indicated that refolded MnP was more closer to native MnP, in terms of its α-helix and β-sheet contents, than soluble MnP. Thus, it appears that the optimized in vitro refolding conditions helped MnP to refold into its native conformation. Nevertheless, soluble MnP exhibited a lower α-helix content than refolded MnP, while its β-sheet content was higher, indicating that the two structures differed. Hence, the lower specific activity of soluble MnP could be ascribed to a change in protein structure, and the lower R_Z_ value could result from an insufficient incorporation of exogenous hemin. Fluorescence spectra also showed the differences in protein conformation. Compared with refolded MnP, soluble MnP exhibited a 4-nm red shift, implying that the tryptophan residue in the protein was more exposed to a hydrophilic environment (MnP contains one tryptophan residue, GenBank accession no. AGO86670.2). Raman spectroscopy is also an efficient technique that is capable of investigating protein molecular structures, good for solid samples as well as aqueous solutions [24]. Recently, Raman spectra have been used for the analysis of different structures of various proteins [24, 52, 53]. Therefore, in the present study, comparisons of major Raman scattering bands were made between soluble and refolded MnP. The results indicated that both enzymes have different Raman characteristic bands, which reflected their structural differences in protein backbone conformations and protein side chains. Moreover, the results from SEC and DLS also supported these findings. The elution profile of refolded MnP produced a main fraction, corresponding to monomeric protein with an Rz value greater than 3. This data (Rz > 3) is a significant characteristic of highly purified MnP and almost identical to that of the native fungal MnP [12]. Further, we confirmed the monomeric nature of the native MnP from *Irpex lacteus* F17 in solution by DLS studies (Additional file 1: Figure S1). Therefore, it was obvious that the quality of refolded MnP was good and the optimized refolding conditions in vitro were achieved. However, we noticed that soluble MnP showed aggregate behavior through dimerization and polymerization in solution. As such, it can be considered that a decrease in enzymatic activity and Rz value could be ascribed to the aggregate formation of soluble MnP. In addition, the DLS results provided reasonable explanation for their differences of CD, fluorescence and Raman spectra between soluble and refolded MnP. Nevertheless, the mechanism of such aggregate formation for soluble MnP still needs further study. Chihi et al. [54] demonstrated that the hydrophobic interactions and the formation of new disulfide bridges resulted in soluble protein aggregates; while, Wang et al. [55] emphasized the importance of the hydrophobic interaction in promoting the protein aggregate, as compared with disulfide bonds. Therefore, we will shed light on the intriguing mechanism of polymerization for soluble MnP in the future.

Although secretory production of soluble MnP in *E. coli* did not result in an ideal enzyme activity, it has some advantages over inclusion bodies [36]. Furthermore, several strategies for the efficient secretory production of recombinant proteins in *E. coli* have been explored [16, 19, 34]. To our knowledge, ours is the first report of soluble MnP expression in *E. coli*, and it provides an optional solution for obtaining biologically active forms of recombinant proteins.

## Conclusions

In summary, using a one-factor-at-a-time method and an orthogonal design, the optimal conditions for the production of refolded and soluble MnP were obtained. The refolded protein yield was improved, and the specific activity of refolded MnP increased by approximately twofold. Soluble MnP was also successfully induced and expressed in *E. coli*, but it was less active than refolded MnP. This phenomenon was confirmed by biochemical and spectroscopic characterizations, as well as their different abilities to decolorize azo dyes. Based on SEC and DLS analyses, refolded MnP was a monomer in solution, while soluble MnP behaved as the protein aggregates. Further studies to improve the activity of soluble MnP and clarify its aggregation mechanism are required and are currently in progress in our laboratory.

## Methods

### Chemicals

Lysozyme, glutathione (GSSG), dithiothreitol (DTT), H_2_O_2_, 2,6-dimethoxyphenol (DMP), 2,2’-azino-bis(3-ethylbenzothiazoline-6-sulfonic acid) (ABTS), phenylmethanesulfonyl fluoride (PMSF), and isopropyl-β-D-thiogalactopyranoside (IPTG) were purchased from Sigma-Aldrich (St. Louis, MO, USA). Triton X-100, Tween-80, ethanol, and other chemicals were obtained from Macklin (Shanghai, China) and were of analytical grade.

### Host strain and expression vector

The *E. coli* strain Rosetta (DE3) was used as a host for recombinant MnP production. The mature MnP cDNA sequence, named *imnp*, was amplified by polymerase chain reaction using the genomic DNA of *I. lacteus* F17 as a template [22]. The *imnp* cDNA was excised with *Xho*I and *Nco*I, and inserted into the expression vector pET28a(+). The resulting construct (named pET28a-*imnp*) was used to transform *E. coli*.

### MnP activity assay

MnP activity was determined by the oxidation of Mn^2+^ to Mn^3+^ at 25 °C and 240 nm (ε = 6500 M^−1^cm^−1^). Incubation mixtures (1 ml) contained 0.11 M sodium lactate buffer (pH 4.5), 1 mM MnSO_4_, 0.1 mM H_2_O_2_, and 25 μl of enzyme sample. One unit (U) of MnP activity was defined as the amount of enzyme needed to oxidize 1 μmol of Mn^2+^ per min.

Additionally, when screening parameters that influence enzyme activity, MnP activity was also estimated using the naked eye to detect a color change based on the oxidation of 2,6-dimethoxyphenol (2,6-DMP) by MnP, which forms a quinone dimer at 469 nm. The DMP oxidation assay was conducted at 30 °C in 0.1 M sodium lactate buffer (pH 4.5) containing 1 mM MnSO_4_, 0.1 mM H_2_O_2_, 1 mM DMP, and 25 μl of MnP. MnP activity assay was performed in triplicate. All the data are the mean values of triplicates.

### Expression of MnP as inclusion bodies

*E. coli* Rosetta (DE3) harboring the recombinant vector pET28a-*imnp* was grown at 37 °C, and recombinant MnP was expressed in the form of inclusion bodies. In vitro refolding was performed according to the method of Chen et al. [22]. The optimal refolding conditions were further studied in small-scale experiments. To begin with, a one-factor-at-a-time method was used to screen influencing factors and corresponding levels on MnP refolding. In this set of experiments, parameter ranges in the refolding solutions were set at: urea (0.1–4.0 M), GSSG (0–5.0 mM), glycerol (0–25%), MnSO_4_ (0–5.0 mM), KCl (0–100 mM), CaCl_2_ (0–300 mM), and hemin (0–500 μM), as well as pH (7.0–9.5) and incubation time (0–110 h). Factors exhibiting significant effects were selected out by observing DMP oxidation in 48-deep well plates. To achieve a higher refolding efficiency and test mutual interactions of those factors obtained by the single-factor experiment, an orthogonal experimental design was used to determine the optimum combination of significant factors and their concentrations. The refolding efficiency was estimated by analyzing the specific Mn^2+^ oxidizing activity of the dialyzed refolded MnP.

### Expression of soluble MnP

*E. coli* cells harboring pET28a-*imnp* were grown at 37 °C in Luria–Bertani broth containing 50 μg/ml kanamycin sulfate and 34 μg/ml chloramphenicol, as described by Chen et al. [22]. When the optical density at 600 nm reached 0.5–0.6, 0.3 mM IPTG was added to the cultures. Simultaneously, different concentrations of chemicals were also added to the culture medium. Then, the growth temperature was lowered from 37 °C to 16 °C, and the cultures were incubated for 16 h, with shaking at 120 rpm, to induce the expression of soluble MnP. Bacterial pellets were obtained by centrifugation at 4 °C at 7800 *g* for 10 min, and they were resuspended in lysis buffer (containing 5 mM imidazole, 0.02 mM PMSF, 0.5 M NaCl, 20 mM Tris, and pH 8.0) in 1/10 of the original culture volume. Then bacteria were lysed by sonication for 30 min. After that, the lysed cells were centrifuged at 4 °C at 10,000 *g* for 30 min, and then, 10 μM hemin and 25 mM CaCl_2_ were added to the supernatant, which was stored at 4 °C for 6 h. Finally, the solution containing soluble MnP was centrifuged at 4 °C for 30 min to remove any excess hemin.

Like the optimization design for the refolding of inclusion bodies, several chemical additives (Triton X-100, Tween-80, ethanol, glycerol, sorbitol, L-proline, and glycine), as well as their concentrations, were tested using the one-factor-at-a-time method to achieve the soluble expression of MnP. Factors exhibiting significant effects were selected by observing DMP oxidation in 48-deep well plates. Subsequently, the optimum combination of significant factors, as well as their concentrations, was further optimized by an orthogonal experimental design to obtain greater soluble MnP expression. The optimum factor combination was estimated by analyzing the specific Mn^2+^ oxidizing activity of MnP after adding exogenous hemin and Ca^2+^ to the cell lysates.

### Purification of recombinant MnP

Recombinant MnP was purified using a Ni^2+^-affinity column (Sangon Biotech, Shanghai, China) according to the manufacturer’s instructions. Sodium dodecyl sulfate-polyacrylamide gel electrophoresis (SDS-PAGE) was conducted using a 12% Tris–HCl separation gel and a 5% Tris–HCl staking gel, followed by staining with Coomassie Brilliant Blue R-250.

### Biochemical characterization of MnP

#### Determination of kinetic parameters of soluble and refolded MnP

The *K*_*m*_ and *k*_*ca*t_ for Mn^2+^ were determined by the hyperbolic, non-linear least squares method at varying MnSO_4_ concentrations (0.025–1.0 mM) in the presence of 0.1 mM H_2_O_2_. The *K*_*m*_ and *k*_*ca*t_ for H_2_O_2_ were determined at varying H_2_O_2_ concentrations (0.01–0.15 mM) in the presence of 1 mM MnSO_4_. The reactions were conducted at 25 °C in 0.11 M of sodium lactate buffer (pH 4.5)_._

#### Effect of pH and temperature on the activity and stability of MnP

The optimum pH for activites of soluble and refolded MnP was evaluated at 25 °C in 0.11 M citrate-phosphate buffer (pH 2.2–8.0) or Tris–HCl buffer (pH 8.6–9.0). pH stability was determined at 25 °C by incubating the enzyme at different pH values (2.2–9.0) for 6 h. The residual MnP activity was measured according to the method used for the MnP activity assay.

The optimum temperature for MnP activity was measured from 10 to 75 °C in appropriate increments in 0.11 M sodium lactate buffer (pH 4.5). To evaluate the thermal stability of MnP, the enzyme was incubated at temperatures ranging from 15 to 65 °C in appropriate increments for 6 h. The residual MnP activity was measured according to the method used for the MnP activity assay.

#### Substrate specificity

The oxidation of guaiacol (*ε* = 12,100 M^−1^ cm^−1^), DMP (*ε* = 49,600 M^−1^ cm^−1^) and ABTS (*ε* = 36,000 M^−1^ cm^−1^) was measured at 456, 469, and 420 nm, respectively, to estimate the substrate specificities of soluble and refolded MnP. The reaction mixtures contained 0.11 M sodium tartrate (pH 4.5), 0.1 mM H_2_O_2_, 25 μl of MnP, with or without 1 mM Mn^2+^, in a total volume of 1 ml. The Mn^2+^ oxidizing activity of both soluble and refolded MnP was 530 U/L.

### Decolorization of Acid Red 18 and Orange G by recombinant MnP

Dye decolorization was measured spectrophotometrically at 506 and 478 nm (WFV UV-2100 spectrophotometer, UNICO, Shanghai, China), which are the maximum visible absorbances of Acid Red 18 and Orange G, respectively.

The decolorization reaction system contained sodium tartrate (0.11 M, pH 4.0), MnSO_4_ (1.25 mM), H_2_O_2_ (0.15 mM)), dye (50 mg/L), and MnP (100 U/L) in a total volume of 1 ml. The reaction was initiated by the addition of H_2_O_2_. Control samples, without enzyme, were performed in parallel under identical conditions.

The decolorization efficiency was obtained from the following equation:

$$ \mathrm{Decolorization}\left(\%\right)=\frac{A_0-{A}_t}{A_0}\times 100\% $$

where *A*_*0*_ is the initial absorbance at 506 nm for Acid Red 18 or at 478 nm for Orange G, and *A*_*t*_ refers to the absorbance at 506 nm or 478 nm at reaction time *t*. The data are presented as the mean values of triplicate experiments.

### Spectroscopic characterization of MnP

#### Far-UV circular dichroism (CD) spectra and UV–VIS spectra

The CD measurements were implemented with a MOS-500 CD spectrometer (Bio-Logic, Grenoble, France), with a 1-mm light path cell, at room temperature. The CD spectra were recorded using a 2-mm bandwidth in the far-UV region (190–250 nm). The protein concentration was 0.1 mg/ml in 0.15 M phosphate buffer, pH 6.5. The UV–VIS spectra were obtained in the 200–700 nm region at room temperature using a DU 730 UV spectrophotometer (Beckman Coulter, Brea, CA, USA), and the protein concentration was 0.5 mg/ml in 0.15 M phosphate buffer, pH 6.5.

#### Fluorescence spectroscopy

All fluorescence spectra of enzymes were obtained at room temperature using an F-4500 FL spectrophotometer (Hitachi, Tokyo, Japan) using a cuvette with a 10-mm path length. Excitation and emission bandwidths were set at 5 nm. Fluorescence measurements were performed at a protein concentration of 0.1 mg/ml.

#### Raman spectra

After protein purification, enzyme solution was dried under vacuum freezing condition for 24 h to produce the solid powder samples. Protein samples were prepared and stored at −20 °C. The Raman spectra of soluble and refolded MnP were obtained in the 400 cm^−1^ to 1800 cm^−1^ at room temperature using a InVla-Reflex instrument (Renishaw, London, England) equipped with excitation from the 785 nm line of a semiconductor laser and a charge-coupled-device (CCD) array detector. The laser was focused through an Olympus 50× objective lens on the solid protein samples. The laser power was 8.4 mW, and the resolution was 0.48 cm^−1^. An acquisition time of 30s was used.

#### Size exclusion chromatography and dynamic light scattering

After being purified using Ni-NTA affinity chromatography, protein samples were further purified by a HiLoad ™ 16/60 superdex 200 gel filtration column (10–600 kDa, GE health care, USA) equilibrated with protein buffer (20 mM Tris–HCl (pH 8.0), 200 mM NaCl). Elution was performed with the same buffer at a flow rate of 1 ml/min. Absorbance was monitored at 280 nm. Fractions containing MnP activity peak were pooled and kept at 4 °C to be used for dynamic light scattering studies. Dynamic light scattering (DLS) spectra were obtained at room temperature using an Dynapro-MS800 instrument (Wyatt, Santa Barbara, CA, USA) and high precision cell made of quartz suprasil with 1.5 mm path length. The protein concentration was diluted to a final concentration of 0.1 mg/ml with 20 mM Tris–HCl (pH 8.0) and 200 mM NaCl. Before measurement, all samples were centrifuged at 4 °C at 10,000 *g* for 10 min. The radius and estimated molecular weight were provided by the Dynamics software.

## Additional files

Additional file 1: Figure S1. Protein size analysis of native MnP. (DOCX 267 kb)
Additional file 2: Raw data used for the production of Fig. 6 and Fig. 7. (XLSX 67 kb)

## Abbreviations

ABTS: 2,2’-azino-bis(3-ethylbenzothiazoline-6-sulfonic acid)
CD: Circular dichroism
DLS: Dynamic light scattering
DMP: 2,6-dimethoxyphenol
DTT: Dithiothreitol
*E. coli*: *Escherichia coli*
FPLC: Fast protein liquid chromatography
GSSG: Glutathione
H_2_O_2_: Hydrogen peroxide
*I. lacteus* F17: *Irpex lacteus* F17
IPTG: Isopropyl-β-D-thiogalactopyranoside
MnP: Manganese peroxidase
PMSF: Phenylmethanesulfonyl fluoride
Rz: Reinheitszahl
SDS-PAGE: Sodium dodecyl sulfate-polyacrylamide gel electrophoresis
SEC: Size exclusion chromatography
