# Supplementary material for: Expression of a fungal manganese peroxidase in Escherichia coli: a comparison between the soluble and refolded enzymes
Source: BMC Biotechnol. 2016 Dec 1;16:87. doi: 10.1186/s12896-016-0317-2 (PMC5134096; doi:10.1186/s12896-016-0317-2)
Supplement: Additional file 1: Figure S1. — Protein size analysis of native MnP. (DOCX 267 kb) [file 12896_2016_317_MOESM1_ESM.docx]

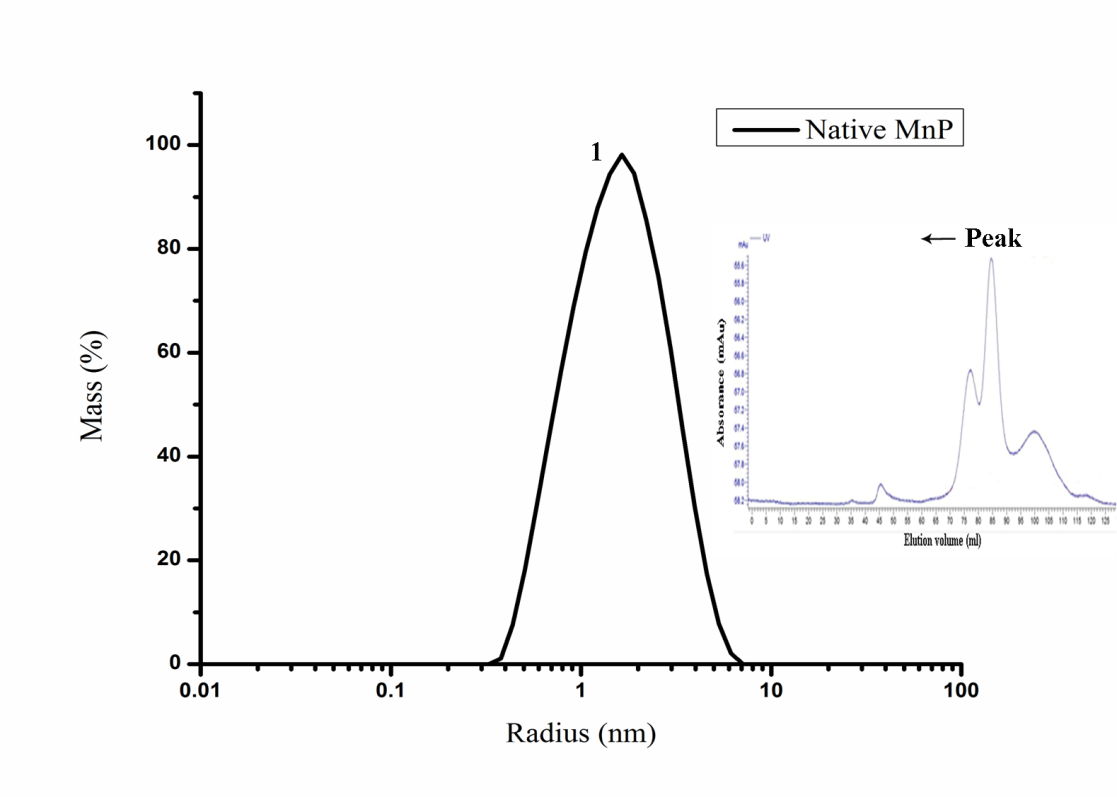


**Figure S1** Protein size analysis of native MnP. Dynamic light scattering result of native MnP from *Irpex lacteus* F17.
